# Uterine Adenocarcinoma with Pulmonary, Liver and Mesentery Metastasis in a Holstein Cow

**DOI:** 10.4061/2010/727856

**Published:** 2009-12-21

**Authors:** George Stilwell, Maria C. Peleteiro

**Affiliations:** Centro de Investigação Interdisciplinar em Sanidade Animal, Faculdade de Medicina Veterinária, UTL, Alto da Ajuda, 1300-477 Lisboa, Portugal

## Abstract

The clinical and pathology features of a cow with uterine adenocarcinoma and multiple metastasis are described. Weight loss, inappetence, mild respiratory signs, and reduced milk yield were evident on clinical examination. Grossly deformed uterus, enlarged iliac lymph nodes, and rosary arranged nodules in the mesentery were felt by rectal palpation. Right side laparotomy revealed numerous small masses covering the omentum, and mesentery. Euthanasia was performed. Necropsy and histopathology exam revealed a uterine adenocarcinoma with multiple pulmonary, liver and mesentery metastasis. Uterine adenocarcinoma with metastasis should be included in the differential diagnosis of cows showing weight loss and mild respiratory distress and palpation of numerous firm nodules in the mesentery should be suggestive of neoplasias' metastasis.

## 1. Case Description

A 5.5-year-old Holstein cow was presented in May 2008 for clinical examination because of inappetence and milk and weight loss over a two-week period. The cow had calved three times and the last calving was in November 2007. In March 2008 the cow was found in heat and was inseminated without difficulty by an experienced stockman.

**Figure 1:**
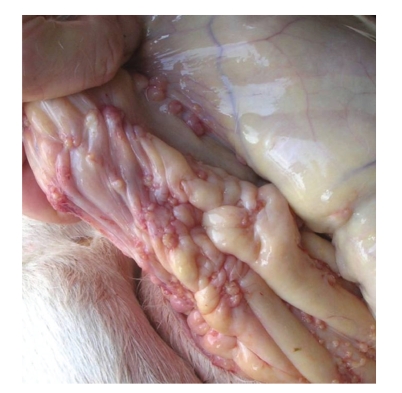
Postmortem examination. Adenocarcinoma metastasis—rosary arranged small size nodules (0.5 to 3 centimetres) in cows' caudal mesentery.

Physical examination revealed rectal temperature, respiratory rate, and heart rate within reference limits. The cow was thin (Body Condition Score 2.5), depressed, ruminal contractions were reduced, and lung auscultation revealed altered sounds especially in the ventral cranial lobes. Teeth grinding were heard occasionally. The left iliac crest was missing as a result of trauma suffered years before but there was no lameness or signs of additional bone lesions. Rectal palpation elicited pain signs and revealed a grossly deformed uterus—the cervix was palpable but cornual and ovarian differentiation was not possible. Introducing a catheter through the cervix was not achievable. Rectal palpation also revealed numerous pea-size round nodules inside the abdomen, arranged as a rosary (Figure 1) and very large and hard iliac lymph nodes. A right side laparotomy revealed nodules covering most of the mesentery and the large omentum, but not the peritoneum. No significant haematological abnormalities were observed except for low red blood cells (4.2 × 10^6^/*μ*L) and leucopaenia (4.5 × 10^3^/*μ*L).

**Figure 2:**
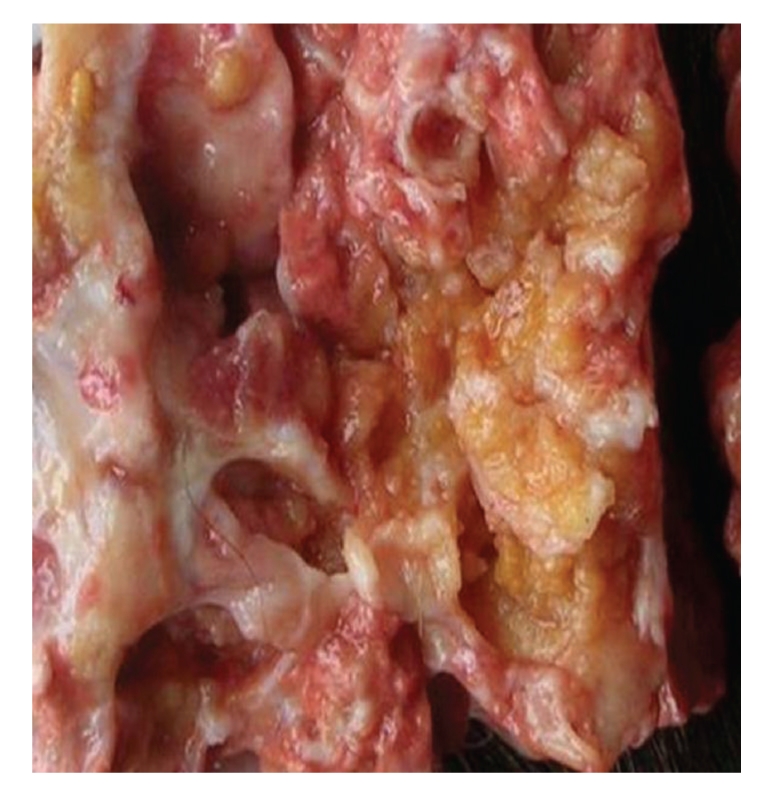
Postmortem examination. Adenocarcinoma metastasis—Two to five centimetres nodules in lung posterior dorsal lobe showing on cut yellow surface.

Euthanasia was performed and postmortem examination showed the uterine body and the proximal part of both horns grossly deformed, irregularly thickened and adherent to the mesometrium, the ovaries and bursa were also adherent to the uterine mass, internal iliac lymph nodes were irregularly enlarged with roughened to nodular surface, numerous nodules were felt throughout the capsular surface of the liver (0.5 to 2 centimetres) and across both lungs' ventral lobes (2 to 5 centimetres), and almost all of the omentum and mesentery, specially in the vicinity of the uterus, was covered in numerous rows of 0.5 to 3 centimetres nodules. There were no nodules in the parietal peritoneum. All the visceral and mesentery nodules were solid and firm in consistency. The pulmonary masses showed on cut section a yellowish surface (Figure 2).

**Figure 3:**
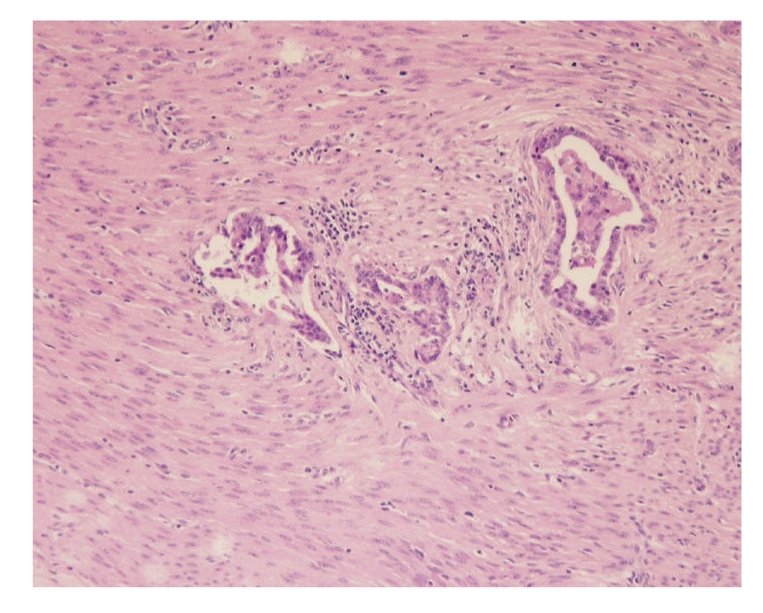
Invasion of lymphatic vessels in the myometrium by cells of the adenocarcinoma (H&E, ×100).

**Figure 4:**
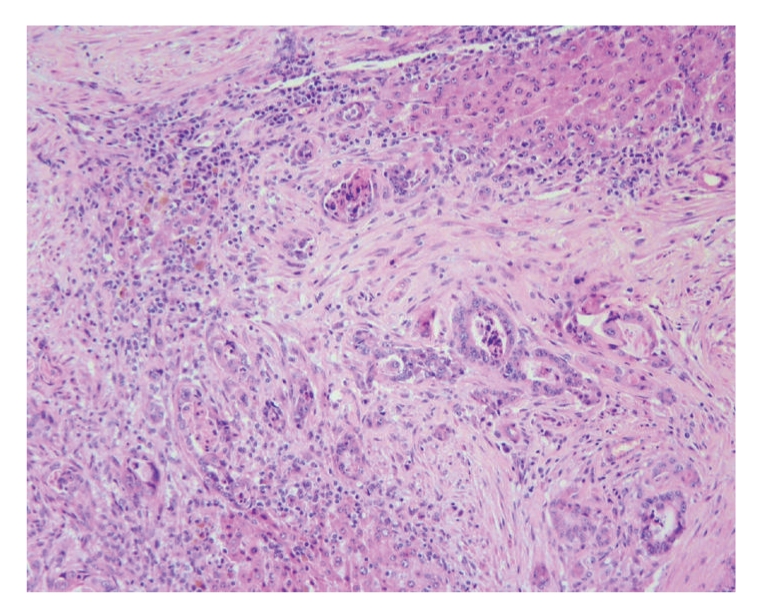
Liver metastasis of uterine adenocarcinoma. The tubular structures infiltrate the interstitial tissue accompanied by dense fibrous tissue and inflammatory infiltrate. Remnants of liver cells can be seen on the left, trapped within the fibrous tissue. Liver lobules are also evident in the upper and lower margins of the image (H&E, ×40).

The histopathology exam showed a highly infiltrative adenocarcinoma characterized by neoplastic cells arranged in tubular structures that soon infiltrate the myometrium and with only a reduced expression in the endometrium. A well-developed stroma, formed by dense fibrous tissue, exceeded the amount of carcinomatous cells. Lymphocyte rich inflammatory infiltrates were frequent. Tumour cells showed medium size, acidophilic cytoplasm and large nucleus with multiple small nucleoli. Anisocytosis and anisokaryosis were marked. Rarely the cells projected into the lumen of the tubular structures forming small papillae with no connective tissue axis. Invasion of lymphatic was obvious in the primitive tumour (Figure 3). Metastasis of the adenocarcinoma were identified in the lung, omentum and liver (Figure 4). Neoplastic cells in the metastasis showed the same characteristics as those in the primitive tumour and the acinar structures were surrounded by well-differentiated fibrous tissue.

## 2. Discussion

Neoplasias in cattle are generally considered rare [1]. Lymphosarcoma is deemed to be the most common neoplasm to affect the uterus of dairy cattle followed by adenocarcinomas [2]. Carcinomas of the endometrium are considered truly rare neoplasmas in domestic animals and not apparently rare due to inadequate postmortem examination [3]. They are more frequent in cattle, when compared to other species, although most cases reported were identified in old cows at slaughter not been common in an autopsy series [4]. In a large histological survey of 302 neoplasms found in 1.3 million slaughtered cattle during one year in 100 abattoirs throughout Great Britain, only 20 were in the female genitalia and all specimens from the body of the uterus (*n* = 5) were smooth muscle tumours [5].

Because it is a malignant tumour that invades lymphatics and veins, uterine adenocarcinoma shows a high capability to spread to distant organs [4–6]. Its invasive capability has been described, with metastasis in the internal iliac and sublumbar lymph node, lungs, and liver [5] as in this case. However, invasion of the omentum has been very rarely reported [6]. The histology of the primitive tumour and its metastasis is in accordance with what has been described for these neoplasms, with the tumour invading the myometrium and showing vigorous fibrous response.

Clinical signs of neoplasia in cattle are not well documented because most reports concern tumours found incidentally at slaughter [1, 2, 7, 8]. The few published clinical studies of uterine adenocarcinoma describe weight loss, pain and subtle respiratory signs suggesting reticulitis or acute pulmonary emphysema and only refer to pulmonary, liver and lymph nodes metastasis [7, 9]. To our knowledge the clinical case described in this paper is the only published report of metastasis also involving the mesentery. This finding may be of clinical importance because mesenteric nodules are easily palpated in contrast with tumours in other organs that are not usually diagnosed in living animals.

Most reports describe uterine adenocarcinomas in cattle as singular tumours and not evident by clinical examination [7–9], but in our case changes were evident by rectal palpation and both horns were involved.

Cows with uterine carcinoma have been found to be 3 to 7 months pregnant [7, 8] or recently calved [9]. In this case the cow had been cycling normally 48 days earlier and the fact that it was inseminated suggests that the tumour was unapparent at that time. This indicates that growth and invasiveness may be very rapid.

The present case is an additional contribution to the very few clinical reports of this neoplasia. Uterine adenocarcinoma with metastasis should be included in the differential diagnosis of cows showing weight loss and mild respiratory distress, independently of age and reproductive status. Palpation of numerous firm nodules in the mesentery is suggestive of neoplasias' metastasis.
